# Surgery

**Published:** 1910-12

**Authors:** A. Rendle Short


### SURGERY.

**Hæmophilia.**—This disease is a remarkable illustration of inheritance by atavism, the general law being that the boys suffer from the disease, but do not transmit, and the girls do not suffer but do transmit. A number of family histories have now been followed for generations, including the Appleton-Swain family in the States, and the Mampel family in Germany, which latter has recently been studied by Lossen,[1] who presents a genealogical tree extending over 100 years, and including five generations with 212 individuals. Sir Almroth Wright[2] gives tables of three other bleeder stocks. The following is a previously unpublished family history obtained by me from a patient living near Bristol:—

Mr. X. = Mrs. X. (nothing known of either).

Children: Daughter (well) | Son (died of bleeding after tooth extraction). | Daughter (well). | Son (probably well).

Children of the second Daughter (well): Four girls (all well).

Children of the first Daughter (well): Son (died, aged 3, of bleeding from a cut finger). | Son (bleeder, covered with bruises, frequent hemorrhages. Died, aged 17). | Son (well). | Daughter (well). | Daughter (well).

Children of the first of these Daughters (well): Girl (well). | Boy (bleeder).

Children of the second of these Daughters (well): Girl (well). | *Boy (the patient).

* The boy in question bled for three days from a cut lip; he had often bled before. The nurse sat him on the edge of the bath to wash him, and a bruise came out across the buttocks.

By combining the published tables it is possible to obtain an answer to several interesting questions. The well-known law of inheritance is thoroughly substantiated in all five series, that is to say, the females transmit to their sons, but do not themselves suffer. The males do not transmit; of eight males in the Mampel family who had male children only one passed on the hemorrhagic diathesis, and the one exception married a first cousin who belonged to the same stock; they had one bleeder son. In one of Wiight's families, however, a hæmophiliac had a normal daughter, but her son was a bleeder.

There are usually a few sons in the afflicted family who escape. My table shows two such. In the Mampel stock only thirty-seven sons out of eighty-two whose mothers transmitted were bleeders, but in Wright's tables only seven escaped out of thirty-one. Moreover, some of the females do not transmit—five out of sixteen in the Mampel family had healthy sons.

Two other questions arise. Do girls ever suffer? And does the disease ever arise *de novo?*

In the published family histories the females certainly seem to escape. Sometimes there is a record of menorrhagia, or post-partum hemorrhage which is probably accidental. But cases are described from time to time which would be regarded as perfectly conclusive of hæmophilia if only the subject had been a boy and there had been bleeders in the family. A little girl who died last year in the Infirmary of dental hemorrhage which nothing would stop may be quoted as an illustration. A very convincing case is given by Squire [3] of a female child who was almost always marked with bruises, who on four occasions bled for days from insignificant abrasions, and on a fifth recurrence of the hemorrhage bled to death from a tiny point inside the mouth. There were no other bleeders in the family. Osler [4] gives instances of cases arising *de novo*, one being in a woman. We had in the Infirmary four years ago a fatal case of hemorrhage from the puncture of a tin-tack inside the cheek in a shoemaker, with no bleeder relatives.

We will barely mention the *symptoms* of hæmophilia, the purpura, epistaxis, hemorrhages, articular effusions, etc. It is, however, worth noticing that in a number of cases the nature or location of the injury is of importance. Pricking the finger to obtain a blood-drop does not usually induce hemorrhage, nor does abstracting it from a vein by means of a syringe; the elastic skin closes over the wound. Some bleeders only pour blood when the injury is above the neck; cuts on the limbs or trunk may produce no more than an ordinary flow. Sir Almroth Wright believes that there are periodical variations in the hemorrhagic tendency. The females in a bleeder family are said to be abnormally fertile.

*Pathology.*—Three deviations from normal have been described in hæmophiliacs.

It has been stated that the blood vessels are abnormally thin (Virchow's hypoplasia of the arteries). In the great majority of instances there is no abnormality in the vessels at all to the naked eye or microscopical examination, and in any case this could scarcely furnish the explanation. The hemorrhage of hæmophilia is a capillary hemorrhage, and the capillary walls could not possibly be thinner than they normally are. The effusion obtained by dry cupping is no greater in hæmophiliacs than in normal people (Morawitz and Lossen).

In the great majority of cases, and perhaps in all, the coagulation time of the blood is much extended. Instead of the normal five to ten minutes, it may be delayed to twenty minutes, one hour, or even longer. Wright gives a number of instances in which a normal time is found in the non-bleeder members of a family, but an extended time in the bleeders. The blood for testing was taken from the finger or from a vein. His observations have been confirmed by Sahli,[5] Addis,[6] and all recent observers. It is true that some writers report no delay in the coagulation time when the blood from the bleeding point is examined, but apart from the many fallacies of all coagulation-time experiments, such as imperfect tubes, varying temperatures, and inclusion of preformed clots, it must also be remembered that the blood always acquires more fibrinoplastic material after severe or prolonged hemorrhage, and hæmophiliacs are no exception to the rule. Indeed, the mouth may often be full of clots, and in many cases the bleeding is eventually checked by coagulation in and about the damaged vessel, though it may be that a dangerous amount of blood has been lost first. Addis shows that the delay in the coagulation time runs parallel with the liability to severe hemorrhage.

Thirdly, the leucocytes are nearly always diminished in number, and particularly the polymorphonuclears. The females of a bleeder family often show this same characteristic.

Perhaps we are not yet in a position to arrive at a diagnosis of the exact cause of the delay in the coagulation time. The conversion of fibrinogen into fibrin takes place under the influence of a substance called thrombin, or fibrin ferment, though it is not really a ferment. This does not exist preformed in the blood, or generalised intravascular thrombosis would take place. It is only formed by the interaction of calcium salts, thrombogen (prothrombin), and damaged cell-nuclei. In ordinary, the last of these is furnished by the cells which are cut through or crushed by the instrument inflicting the injury. And as the endothelial cells lining the blood-vessels are not immortal, and their disintegration would initiate thrombin formation, and thus coagulate the fibrinogen, provision is made that the liver should shed out into the blood an anti-thrombin sufficient to prevent this, but not sufficient to interfere with our defence against external hemorrhage. The thrombogen is probably derived from leucocytes, but is intimately associated with fibrinogen.

Hæmophilia might therefore be due to a defect in the amount either of fibrinogen, of thrombogen, or the material (thrombokinase) supplied by the cell-nuclei, or of calcium salts, or it might be due to an excess of anti-thrombin from the liver. It is certain that the fibrinogen is normal in amount, and there is no evidence of any lack of calcium. The cases in which bleeding from the mouth was excessive, but bleeding from the limbs did not pass beyond normal limits, are in favour of the theory that the cell-nuclei in the danger zone contain too little fibrinoplastic material, but Addis shows that damaged tissue, such as testis, can only very slowly induce clotting in hæmophiliac blood, so that we must look elsewhere for the real explanation.

Indeed, it is not clear that the delay in the coagulation time is due to a deficiency in quantity of any of the ordinary fibrinoplastic elements of the blood, Addis now concludes that the essential trouble is a perversion of the thrombogen, which yields a normal thrombin, but yields it too slowly. Haemophiliac fibrinogen is readily clotted either by normal or haemophiliac thrombin. Washed corpuscles from normal blood immediately clot haemophiliac blood (Sahli, Addis). The blood will clot well where it is in contact with the tissues, but the thrombin which should extend to and coagulate the central parts of the wound acts exceedingly slowly or not at all, so that the peripheral fibrin may get dislodged for lack of reinforcement, and then the bleeding starts again.

*Treatment.*—The women of bleeder families ought not to be allowed to marry, or at least they should not have children. Their male offspring should be wrapped in cotton-wool, figuratively speaking, all their days.

If the ordinary methods of treatment fail (pressure, adrenalin, rest), some of the following special devices should be resorted to.

Weil[7] pointed out that injection of horse serum in a bleeder was followed after forty-eight hours by a diminution in the coagulation time, and the treatment has been a good deal used since. Our experience at the Infirmary is not favourable, and Sir Almroth Wright, thinks it acts too slowly to be of practical value. The most convenient form in which to obtain horse serum is diphtheria antitoxin.

Wright[2] makes three important suggestions :—

(*a*) The administration of calcium lactate or magnesium carbonate or lactate in fifteen grain doses to children, or sixty grains to adults, followed by half-doses daily for three days. He allows that the treatment may fail, and that it may even make matters worse if too large quantities are given. These drugs must not be administered hypodermically, or sloughing results. The causes of failure are twofold: the drug may not get absorbed; or the blood content of calcium may already be the optimum. It is well known that too much calcium delays coagulation.

(*b*) The administration of $CO_2$. This may succeed when calcium salts fail. It may be obtained from a cylinder of liquid $CO_2$, or from a Wolff's or Kipp's apparatus, containing marble and HCl, and only a small stream should be supplied. Dyspnœa should be avoided. Venous blood clots more easily than arterial; this fact is responsible for the introduction of the method.

(*c*) Physiological styptics. Wright regards this treatment as of great value in stopping the flow, but like other styptics, they cannot cause intravascular clotting, so other methods should be adopted as well. The advantage over iron and tannin preparations is that they are painless, and do not cause sloughing or suppuration. Minced thymus with saline (one part thymus to ten parts saline) is the best. It is slower in action than the escharotic styptics, but produces abundant firm clot.

As a prophylactic against hemorrhage, Wright gives thymus tabloids (up to 100 grains daily).

There remains one last resort in the most desperate cases. Goodman[8] has published a well-written, almost dramatic description of his treatment of a child dying of hæmophilia. He was a Jewish boy, aged 2½, a well-known bleeder, with other bleeders in the family. He was admitted to hospital for a cut inside the cheek, which bled slowly but incessantly for two days. The usual pressure, adrenalin, styptics, diphtheria, antitoxin, and calcium salts were tried in vain. Finally the child lay motionless and pallid, the hæmoglobin 12 per cent., and the hemorrhage continuing. Goodman then decided to inject normal human blood. A donor, not a relative, was tested by Wassermann's method, which was negative. Under novocain anæsthesia his radial artery was connected by an Elsberg cannula with the femoral vein of the child. There were some initial difficulties in getting a good flow; hot cloths had to be applied, and finally the basilic vein was substituted for the femoral, on account of differences in the level of the patients. The transfusion continued for twenty-eight minutes. During this time colour gradually mounted up in the cheeks of the little sufferer, the breathing became audible once more, the almost watery blood acquired its normal hue, and when the process was stopped the child's hæmoglobin was 70 per cent. The significant point was that the bleeding was instantly staunched. Both made an uneventful recovery. The donor had to rest for several days.

This interesting case does not stand alone, and obviously no patient should be allowed to die without transfusion in future. Animals' blood will not do for large transfusions, on account of the hæmolytic phenomena. The connection between the two patients may be made by dissecting out under local anæsthesia a few inches of artery and vein respectively, and connecting them either by a short oiled glass cannula or by Carrell's immediate suture. The latter method has been adopted with success for direct transfusion in septicæmia as well as in hæmophilia, but the technical difficulties may be considerable.

When a healthy adult is used to supply blood to an infant, the donor suffers no ill effects, but a period of rest is desirable. In many cases, especially if older children or young adults are to be transfused, it would be well to provide two donors, either consecutively or simultaneously.

REFERENCES.

[1] **Lossen,** *Deutsche Zschr. f. Chir.*, 1905, lxxvi. 1.
[2] **Sir Almroth Wright,** *Allbutt's System of Medicine*, 1909, v. 918.
[3] **Squire,** *Brit. M. J.*, 1910, i. 1168.
[4] **Osler,** *Lancet*, 1910, i. 1226.
[5] **Sahli,** *Ztschr. f. klin. Med.*, 1905, lvi. 264; *Deut. Archiv. f. klin. Med.*, 1910.
[6] **Addis,** *Quart. J. Med.*, 1910, iv. 14; *Brit. M. J.*, 1910, ii. p. 1422.
[7] **Weil,** *Compt.-rend. Soc. de Biol.*, 1909, p. 454.
[8] **Goodman,** *Ann. Surg.*, 1910, lii. 457.

A. RENDLE SHORT.

